# Assessment of index-based traffic noise annoyance level at major road intersections in a tourist city: A case study towards environmental sustainability

**DOI:** 10.1016/j.heliyon.2024.e40005

**Published:** 2024-11-01

**Authors:** Hrithik Nath, Sajal Kumar Adhikary, Saleh Alsulamy, Abdulla Al Kafy, Zullyadini A. Rahaman, Srabanti Roy, Mohammad Iqbal Hossain, Abdulla Al Mamun

**Affiliations:** a Department of Civil Engineering, University of Creative Technology Chittagong (UCTC), Chattogram, 4212, Bangladesh; b Department of Civil Engineering, Khulna University of Engineering & Technology, Khulna, 9203, Bangladesh; c Department of Architecture, College of Architecture & Planning, King Khalid University, Abha, 61421, Saudi Arabia; d Department of Urban & Regional Planning, Rajshahi University of Engineering & Technology (RUET), Rajshahi, 6204, Bangladesh; e Department of Geography & Environment, Faculty of Human Sciences, Sultan Idris Education University, Tanjung Malim, 35900, Malaysia; f Department of Public Health, University of Creative Technology Chittagong (UCTC), Chattogram, 4212, Bangladesh

**Keywords:** Road intersections, Annoyance, Noise index, Mean dissatisfaction score, Cox's bazar

## Abstract

Urban noise pollution poses significant challenges to public health and environmental sustainability, particularly in rapidly developing tourist destinations. Noise pollution and associated annoyance level in five major intersections of Cox's Bazar City, Bangladesh, was assessed in this study during the peak tourist season. Noise measurements were conducted using various indices (L_10_, L_eq_, and TNI) across morning, midday, and afternoon time slots. TNI scores were compared with Mean Dissatisfaction Score (MDS) standards to assess traffic-induced noise annoyance levels. Additionally, a survey of 675 respondents was conducted to assess their perceptions of noise pollution. Statistical analyses included linear regression for noise indices, multinomial logistic regression for TNI-related dissatisfaction, and ordinal logistic regression for respondents' perceived annoyances. Results revealed significant noise pollution issues, with L_eq_ scores consistently exceeding national guidelines across all intersections and time periods, particularly on weekends during afternoon timeslots. TNI values frequently surpassed standard dissatisfaction regulations, with 19 out of 105 time slots exhibiting extreme dissatisfaction levels. Link Road and Kolatoli Circle intersections consistently showed higher noise levels and dissatisfaction. Over 95% of survey respondents perceived increased noise pollution during peak tourist seasons, with 87.11% describing it as "extremely" or "very" noisy. Longer exposure duration and awareness of health risks were significantly associated with reported perceived annoyance levels. Respondents reported various health impacts, including annoyance (84.44%), headaches (62.37%), and cognitive impairment (44.44%). This comprehensive study provides valuable insights for policymakers, city planners, and environmentalists to develop sustainable urban strategies that balance the acoustic environment with the well-being of residents and tourists alike.

## 1 Introduction

Noise pollution, the presence of unwanted sound from human activities, has emerged as a critical global concern, particularly in urban environments. The World Health Organization (WHO) has classified it as a serious public health issue, associating it with various health problems including stress, sleep disturbances, and cognitive impairments [[1], [2], [3], [4], [5], [6]]. Rapid urbanization and increased tourism have intensified this problem [[7], [8], [9]], elevating noise pollution to the third most dangerous factor in major cities [10,11]. The study of noise and associated annoyance is crucial from multiple perspectives. Noise affects not only environmental quality but also resident satisfaction [12], overall well-being, and health [13]. Given these wide-ranging impacts, research into noise pollution is essential for addressing its effects on both individuals and communities [14,15].

Long-term exposure to excessive noise can potentially have more severe consequences, including hearing loss and cardiovascular problems [16,17]. Elevated noise levels are recognized for their capacity to induce annoyance [18]. Numerous investigations have documented that noise-induced disturbance is linked with various adverse outcomes, encompassing negative impacts on mental well-being, heightened feelings of anger, disappointment, and dissatisfaction, as well as a sense of withdrawal, helplessness, and depression [19,20]. It is also associated with higher anxiety levels, increased distractibility, heightened agitation, and overall fatigue [21,22]. Furthermore, there is an established correlation between noise-related sleep disturbances and increased risk factors for hypertension, coronary heart disease, psychological stress, general annoyance, and sleep disturbances in the broader populace [[23], [24], [25]]. Nonetheless, the primary issue linked with prolonged exposure to environmental noise pollution is the development of hearing impairment due to noise, a condition with far-reaching consequences for the welfare of those enduring extended noise exposure, their families, and the broader national context [26,27]. A GIS-based study in Bahadurpur, UP, India, found that 75–92% of individuals near noisy intersections suffered from hearing impairment and other health issues, compared to 13–30% in quieter areas [28]. Additionally, it is worth mentioning that a substantial 80% of individuals experiencing noise-induced hearing loss (NIHL) reside in countries classified as low- and middle-income economies [29,30], similar to Bangladesh [31].

Major intersections, while vital for urban transportation and economic development, are significant contributors to noise pollution. These critical nodes represent microcosms of a city's soundscape, where vehicular traffic, honking horns, and the hubbub of tourist activities converge. The growing traffic density at these junctions often leads to elevated noise levels, potentially harming both human health and the environment [[32], [33], [34]]. The acoustic challenges at many of these intersections are exacerbated by factors beyond traffic alone, such as announcement systems, vibrant commercial activities, ongoing construction, and ambulance sirens. It is essential to also account for the noise generated by these additional sources [35]. Given the concentration of urban activities at intersections and their potential impact on the acoustic environment, it is imperative to measure and evaluate noise levels, particularly during peak tourist seasons [34,36]. Determining noise pollution at intersections during tourist seasons is crucial as it captures peak urban activity levels [37]. This period typically sees increased traffic, additional noise sources from tourist-related activities, and potentially higher short-term impacts on health and environment [38,39]. Understanding the temporal fluctuations of noise pollution throughout the day during the tourist season is essential for developing effective mitigation strategies and implementing noise control policies. Comparing these measurements with normal times provides valuable insights for urban noise management and planning. These efforts are crucial for improving the overall urban soundscape and addressing the adverse effects of noise at these bustling crossroads.

Urban noise pollution, particularly around intersections and roundabouts, has been extensively studied in various global contexts. Research in Khulna, Bangladesh, revealed that noise levels at major traffic intersections exceeded acceptable thresholds, resulting in significant health impacts such as annoyance, headaches, and hearing loss among residents [40]. Similarly, a study in Doha, Qatar, found that daytime traffic noise levels at urban road intersections consistently surpassed local and WHO guidelines, with weekday and weekend averages ranging between 67.6 and 77.5 dB(A) and 68.8–76.9 dB(A), respectively [41]. Comparing noise pollution at signalized intersections and roundabouts, research has yielded mixed results. One study in an urban environment found that roundabouts, especially two-lane configurations, produced higher traffic noise levels than signalized intersections, despite similar or lower traffic volumes [42]. Conversely, a comparative study using microscopic traffic simulation and dynamic noise tools suggested that signalized intersections can emit more noise than roundabouts due to vehicle acceleration, although roundabouts may become noisier under congestion [43]. Another study employing dynamic traffic noise simulation found that noise pollution peaks at saturation, with exit lanes contributing significantly more noise energy than entrance lanes [44]. Research on signalized roundabouts indicated that noise levels (LA_eq_) were 2.5–10.8 dB higher compared to classic signalized intersections and 3.3–6.7 dB higher than at non-signalized roundabouts, with variations attributed to intersection geometry, traffic type, and heavy vehicle volume [45]. It's important to note that noise pollution at busy intersections can be further exacerbated by other anthropogenic factors. These may include public address systems or "miking" for announcements or advertisements, bustling crowds in nearby areas, and the presence of markets or bazaars. Such additional sources of noise can significantly contribute to the overall soundscape, potentially increasing the complexity and intensity of noise pollution at these urban focal points. Neighborhood noise, those close to or around the intersections, can also play a substantial role in urban soundscapes. This includes construction activities, human-generated sounds, noise from neighbors, sirens, religious temples, nightclubs, domestic appliances, and even animals [[46], [47], [48]]. In Skopje, Macedonia, for instance, 75% of respondents identified construction and other human activities as the most annoying noise sources, underscoring the importance of addressing non-traffic related noise [48]. A past investigation into urban dwellers' annoyance from traffic noise found that street layout and types of public transport affect how residents respond to noise. Specifically, wider streets (L-shaped) led to higher annoyance compared to narrower streets (U-shaped), even when the noise levels were the same [49].

The annoyance caused by noise has been a subject of extensive research, revealing its significant impact on urban residents' well-being. Studies have consistently shown that traffic-related sound sources receive higher annoyance ratings compared to nature-related sounds at comparable noise levels [50,51]. The Canadian Perspectives on Environmental Noise Survey found that factors like sleep disturbance and noise sensitivity were key predictors of high noise annoyance from various sources [12]. A cross-sectional study near a super specialty hospital found that traffic noise significantly increased annoyance and poor sleep quality, despite high literacy rates among residents [18]. Research using advanced modeling techniques, such as Structural Equation Modeling (SEM) and Artificial Neural Networks (ANN), has identified factors influencing noise-induced annoyance, including sensitivity, exposure hours, profession, sleeping disorders, and education [[52], [53], [54]]. These models have achieved prediction accuracies ranging from 68.5% to 71.2%. Studies in various urban areas, including São Paulo, Brazil, and Denmark, have found high percentages of adults annoyed by road traffic noise, with strong correlations between noise sensitivity and annoyance [55,56]. Furthermore, research has linked traffic noise annoyance to increased use of psychotropic medications, particularly anxiolytics and antidepressants [57]. A study in Bhubaneswar revealed that noise exposure leads to increased annoyance, higher blood pressure, and various psychophysiological issues in motorcycle riders [54]. Laboratory experiments have also shown that the temporal pattern of noise affects annoyance levels, with longer quiet periods and more regular breaks reducing annoyance, although cognitive performance remained largely unaffected [58]. Comprehensive mitigation measures, particularly traffic management, site surveillance, and temporary noise barriers, can help reduce highway construction noise annoyance, with residents generally satisfied despite a perceived decrease in effectiveness over time, while a previous study reported that annoyance from noise in a large road construction project was primarily influenced by socio-demographic, psychosocial, and contextual factors rather than the noise levels themselves [59,60].

While previous studies have examined noise pollution in urban areas of Bangladesh [40,61], a gap was identified in the literature regarding traffic-induced noise and annoyance at road intersections during tourist seasons. This study was designed to address this gap by focusing on Cox's Bazar City during its peak tourist months (November to February). The principal research objectives were established as follows: (1) to assess noise pollution levels and patterns at major road intersections and compare them with national standards; (2) to quantify noise-induced annoyance in terms of dissatisfaction; and (3) to evaluate local residents' and workers' perceptions of noise and associated annoyance through a questionnaire survey. A combination of noise level monitoring techniques and sophisticated data analysis was employed to examine diurnal and temporal fluctuations in noise levels. Furthermore, an observation-based cross-sectional survey was conducted to provide insights into noise exposure, perceived intensity, peak periods, health impact awareness, and identified noise sources. The findings from the study can expand knowledge on noise pollution and inform targeted interventions by urban planners, policymakers, and environmentalists, ultimately balancing development with resident and tourist well-being in Cox's Bazar and similar destinations.

## 2 Methods

### 2.1 Description of study area and context of noise pollution in Cox's bazar city

Cox's Bazar City, a renowned tourist destination in the southeastern coastal region of Bangladesh, serves as the study area for investigating noise pollution during the tourist season. Bordered by the Bay of Bengal to the west, the Naf River and Myanmar to the south, and the Chattogram Hill Tracts to the east, Cox's Bazar has undergone significant development to support its thriving tourism industry. The city is famed for its picturesque landscapes, including the world's longest natural seashore, rolling hills, and lush green forests. These natural attractions, along with its rich cultural heritage and historical sites, have established Cox's Bazar as a major tourism hub, attracting millions of local and international tourists annually, particularly during the peak tourist season [62].

Cox's Bazar's appeal lies in its potential for adventure sports, water activities, and relaxation, drawing tourists seeking diverse experiences. Annually, the city welcomes approximately 840,000 foreign tourists and 13,700,000 domestic tourists. This influx drives demand for local goods and services, creating a multiplier effect throughout the regional economy. According to the Bangladesh Government, tourism directly contributed 296.6 billion BDT to GDP (1.90% of total GDP) and supported 19 million jobs (3.6% of total employment) in 2014. Projections for 2025 suggest that tourism's contribution to GDP will reach 566.3 billion BDT, generating employment for approximately 24 million individuals [[63], [64], [65]]. Thus, Cox's Bazar's prominence as a major tourist destination not only promotes cultural exchange and understanding but also bolsters the local economy by generating substantial revenue [66,67].

The tourist season in Cox's Bazar typically extends from November to February, characterized by mild and pleasant weather. During this period, the city experiences a surge in tourists, leading to a significant increase in tourism-related activities and services [68]. However, this bustling tourism activity also brings potential challenges, notably noise pollution. Numerous news articles have reported rising noise pollution within Cox's Bazar City [69,70]. Increased human activity, vehicular movement, and entertainment venues contribute to elevated noise levels, which can detract from the tranquil ambiance that tourists seek. Noise pollution, coupled with overcrowding and related inconveniences, may deter potential visitors and diminish the tourism sector's attractiveness. It also poses a nuisance for residents, who may struggle with sleep disturbances and concentration issues. This highlights the need for comprehensive research to understand the extent of noise pollution, its impact on tourism, and potential mitigation strategies. Addressing these concerns through strategic planning and sustainable development measures is essential to maintain Cox's Bazar City as a coveted tourist destination and ensure its long-term economic vitality.

**Fig. 1:**
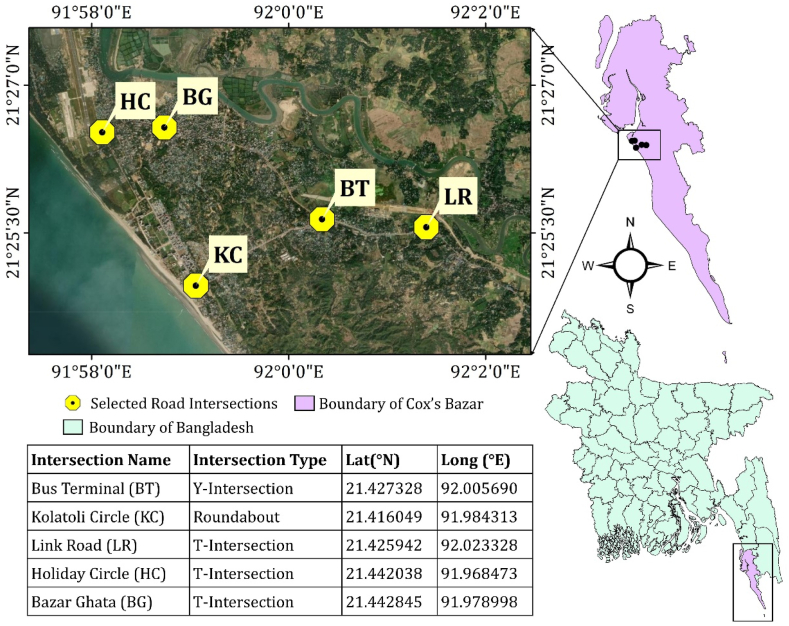
Map of study area with locations of selected traffic intersections.

To accommodate the growing number of visitors, hotels, resorts, restaurants, and various entertainment facilities have been established along the coastline. During the peak season, the city experiences a surge in vehicular traffic, particularly at major road intersections, leading to elevated noise pollution levels. This study focuses on five major road intersections within Cox's Bazar City for the noise pollution analysis: Bus Terminal (BT), Kolatoli Circle (KC), Link Road (LR), Holiday Circle (HC), and Bazar Ghata (BG) (Fig. 1). These intersections were selected based on criteria such as traffic density, proximity to popular tourist attractions, and relevance to the overall traffic flow within Cox's Bazar. Consequently, the chosen intersections represent the typical traffic conditions experienced during the tourist season.

### 2.2 Calculation and analysis of noise indices

For environmental noise monitoring, such as road traffic or community noise evaluations, statistical noise percentile levels (L_N_ [dB]) are frequently utilized. Percentile noise levels (L_N_ [dB]) indicate the sound level surpassed by N% of the total measurement period. In the current study, L_10_ was considered as one of the parameters for noise exploration, due to its frequent employed to indicate the upper limit of fluctuating noise, such as that generated by road traffic. L_90_ is generally regarded as the background noise level. At the midpoint of the entire noise observation, L_50_ has been included in certain community noise assessments in several countries [71]. Noise climate is the range over which sound levels fluctuate over a specific interval, which can be expressed by Eq. (1), where, L_10_ and L_90_ are the noise levels exceeded for 10% and 90% of the time of the measurement duration, respectively [72].

(1) $$\text{NC}=L_{10}\;–{\;L}_{90}$$

Equivalent continuous noise level, commonly being denoted as L_eq_, plays a pivotal role in acoustic analysis. This metric represents the consistent noise level that would generate an equivalent total sound energy output over a specified time interval. It means a changing sound source as a single number and considers both the intensity and length of all sounds occurring during the given period. Its significance lies in its ability to understand potential hearing damage risks and noise-related grievances comprehensively. L_eq_ is the central parameter in most noise studies, and many community noise regulations rely on L_eq_ as the legal standard for managing and controlling noise levels within communities [40,73,74]. In the current study, the L_eq_ measurements for three specific time durations in a day (morning, midday, and afternoon) were computed using Eq. (2), where L_50_ is the noise level exceeded for 50% of the time of the measurement duration, and NC is Noise Climate.

(2) $$L_{\text{eq}}=L_{50}+\frac{{\text{NC}}^{2}}{56}$$

As L_eq_ is an insufficient descriptor of the annoyance caused by fluctuating noise [75], the traffic noise index (TNI) was used to evaluate and quantify the annoyance associated with noise levels produced by vehicular traffic. It is a widely used and effective tool for assessing and controlling noise pollution in cities and along highways. The index considers continuous noises generated by vehicles on the road, intermittent noise resulting from acceleration and deceleration, and the impact of excessively loud vehicles compared to regular traffic noise. TNI (expressed in dB) was calculated using Eq. (3) [76].

(3) $$\text{TNI}=4\text{NC}+L_{10\;}–\;30$$

While L_10_ has been utilized in some studies to measure noise effects on health and quality of life [59,60,77], this investigation primarily employed TNI and L_eq_ as more comprehensive indicators for noise pollution assessment. The Traffic Noise Index (TNI) is particularly applicable to this analysis due to its capacity to quantify annoyance associated with fluctuating noise levels in urban traffic environments [78,79]. TNI presents several advantages over L_10_ for traffic-related noise annoyance assessment. It incorporates both background noise (L_90_) and peak noise levels (L_10_), thus providing a more holistic representation of the noise environment [80,81]. Furthermore, TNI accounts for noise level variability, which is critical for understanding the impact of intermittent loud noises typical in traffic scenarios [80]. Additionally, L_eq_ has been found to correlate better with long-term effects of noise exposure on health and well-being compared to single-event metrics like L_10_ and L_50_ [82,83]. Its wide acceptance in noise regulations and guidelines facilitates comparison with established standards [84,85].

### 2.3 Noise standards and assessment of annoyance level

In Bangladesh, the Department of Environment (DoE) implemented the Noise Pollution Control Rules of 2006 [85], as outlined in Section 20 of the Bangladesh Environment Conservation Act of 1995. The primary objective of these regulations was to establish precise directives concerning noise pollution and the permissible noise levels across various zones. Comparable guidelines were also furnished by several international entities, such as the American Association of State Highway and Transportation Officials (AASHTO) and the Federal Highway Agency (FHA), all sharing the common goal of noise pollution mitigation. Table S1 presents the acceptable daytime (06:00 a.m. to 09:00 p.m.) noise levels (L_eq_) on the dB scale, categorized into five distinct types of zones: silent areas, residential areas, mixed areas, commercial areas, and industrial areas. Within the context of this research endeavor, an attempt was made to assess the noise levels at designated intersections against the L_eq_ (dB) standards specified by the aforementioned regulatory bodies.

Numerous studies involving annoyance as an outcome of noise pollution can be widely found in literature [[86], [87], [88], [89], [90]]. Standard noise indices, viz., L_eq_, L_max_, L_np_, TNI, and others, have been used by various researchers and institutions around the world to quantify annoyance due to traffic-generated noise [91]. For the measurement of assessing community annoyance using traffic noise data, MDS is a previously developed five-point scale to predict noise-generated annoyance extensively used in acoustic studies [92,93], which is described in Table S2. Using regression analysis, the scale is correlated with various noise indices by pairing mean dissatisfaction scores (MDS) with different noise parameter values [93]. The scale has the lowest threshold of 1 for 'negligible' mean dissatisfaction, 3 for ‘moderate’ dissatisfaction, and a maximum value of 5 for 'extremely' annoying dissatisfaction levels. Any MDS of 4 or higher is prohibitive, and an MDS greater than five may have negative health consequences in terms of psychological and physiological damage. In this study, the level of annoyance at all the intersections in different time slots was obtained using TNI and associated mean dissatisfaction scores (MDS).

### 2.4 Formulation of survey questionnaire

In the current study, a field survey was performed by distributing a printed questionnaire (Fig. S1) to individuals in addition to the assessment of noise levels. The primary intention of the survey was to gather data about the awareness and perspectives of the general populace concerning noise pollution at the five designated intersections. The questionnaire was meticulously developed based on a comprehensive review of existing research [49,92] and relevant standards [[94], [95], [96]]. The questionnaire consisted of twelve questions, primarily exploring qualitative aspects of noise perception and its correlates. The questionnaire was structured to cover four specific criteria: (i) respondent demographics, (ii) residential characteristics, (iii) knowledge of noise-related issues, and (iv) perceptions of noise pollution and annoyance. The question on respondents’ perception towards annoyance was: “Thinking about the last 12 months or so, when you are at the intersection, how much does noise from the intersection annoy you during the tourist season?”. To ensure accurate and reliable data collection from a population with limited English proficiency, a questionnaire adapted to the local language and cultural context was essential [95]. The questionnaire was therefore translated into Bengali, the local language, with careful consideration given to question phrasing and structure to maintain clarity and avoid cultural misunderstandings. The questionnaire form was finalized following a pilot survey consisting of 20 respondents to make sure that the questions were clear and easy to read.

### 2.5 Noise and survey data collection

**Fig. 2:**
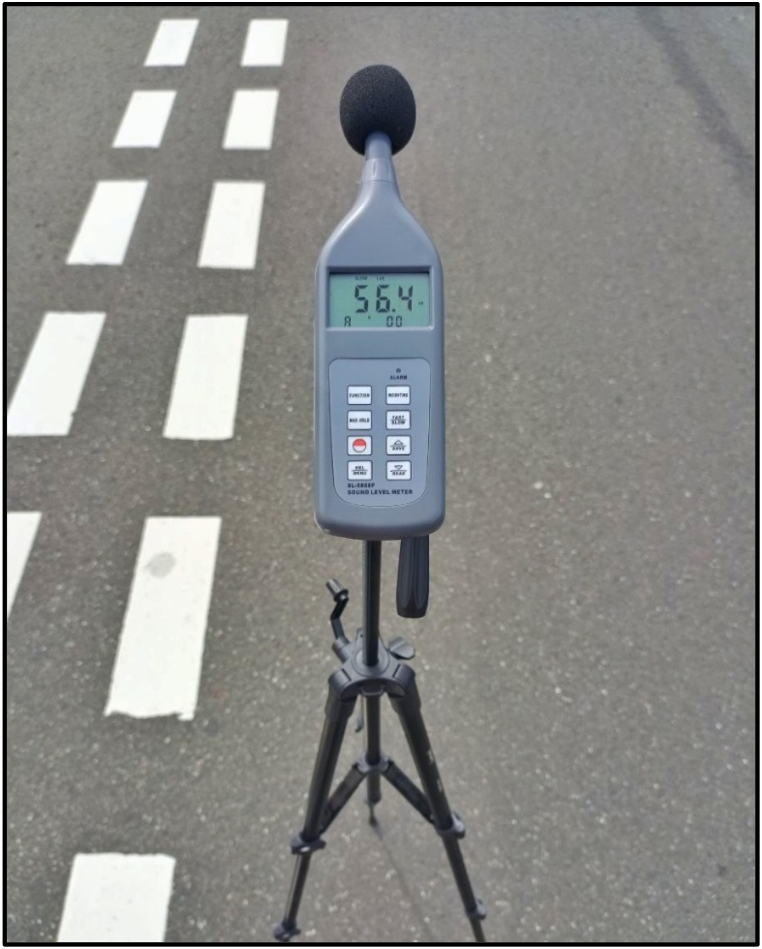
Noise monitoring installation at the intersection.

In the present study, noise level data were collected from five distinct intersections, as illustrated in Fig. 1, to investigate road noise in dB. A Digital Sound Level Meter SL-5868P was employed for data acquisition. This device offers a measurement range of 30–130 dB with an accuracy of ±1.5 dB, featuring A, C, and Z frequency weighting options and Fast/Slow time weighting settings. The SL-5868P's data logging capabilities facilitated continuous monitoring of noise levels over extended periods, ensuring comprehensive data collection for analysis. To mitigate potential interference from obstacles such as parked vehicles, vegetation, or uneven ground surfaces that could introduce distortions to the noise measurements, the receiver was positioned at a height of 1.5 m, as illustrated in Fig. 2, adhering to predefined guidelines for road noise measurements [97,98]. This height was carefully selected as a compromise to balance the capture of representative traffic noise while minimizing the impact of ground effects. It was ensured that no obstacles were present between sound sources and microphones during measurements, thereby guaranteeing reliable data collection.

The data collection process involved capturing noise measurements at regular 5-min intervals over a 12-h period, from 8 a.m. to 8 p.m., encompassing all weekdays from November 01, 2023, to February 29, 2024, when the tourist activities are generally at the peak. For analytical purposes, each day was segmented into three distinct time slots: morning (8 a.m.–12 p.m.), midday (12 p.m.–4 p.m.), and afternoon (4 p.m.–8 p.m.). This approach allowed for a comprehensive exploration of noise patterns and variations throughout the designated periods. Throughout the data collection process, particular attention was given to continuous noise sources, such as microphones, soundboxes, and large gatherings. These sources were considered integral to the overall noise environment of urban settings, contributing significantly to the complex soundscape. The portability and user-friendly interface of the SL-5868P facilitated efficient on-site measurements, ensuring accurate and reliable data collection across all identified noise sources and environmental conditions. This methodological approach, combining advanced noise measurement technology with strategic sampling techniques, enabled a thorough and precise assessment of the urban noise environment across the selected intersections.

To gather responses to the survey questionnaire, printed documents containing the survey questions were distributed among individuals at the road intersections, and subsequently, the completed questionnaires were collected from the respondents. The minimum sample size was obtained by using Eq. (4) [99].

(4) $$n=\frac{p\times \left(100‐p\right)\times z^{2}}{E^{2}}$$

Here, n is the minimum number sample size, p is the effect size which was considered 0.5 (medium effect size), z is the z-score corresponding to a 95% confidence level which is 1.96, and E is the maximum error allowed which was 5%. The formula yielded 385 as the minimal sample size. Thus, more than 700 individuals were approached in the field survey. The survey respondents were selected from a diverse pool, including individuals such as professionals, rickshaw pullers, automobile drivers, receptionists, traffic enforcement personnel, tradespeople, street vendors, manual laborers, students, educators, and healthcare practitioners, all of whom have completed at least primary level of education and typically spend significant durations of weekdays in the vicinity of these intersections. Importantly, the selection criteria for the survey specifically targeted individuals who spend moderate to considerable amounts of time in and around the intersections. This criterion ensured that the survey captured perspectives from those most frequently exposed to the area's noise conditions. It is important to mention that the questionnaire forms were not randomly distributed; instead, respondents were carefully monitored to ensure that they completed each question thoughtfully and accurately, promoting meaningful responses. Some respondents were excluded during the survey due to communication barriers, false answers, or lack of seriousness, as well as personality or behavioral factors that could compromise data quality or respondent cooperation. The survey responses were collected between November 1, 2023, and February 29, 2024, alongside noise data collection. Following a meticulous review, responses that were incomplete or contained inadequate information were excluded from the dataset. Ultimately, 675 valid responses were retained for further analysis.

### 2.6 Outcome and predictor variables, and statistical analysis

For the assessment of noise pollution and associated annoyance, the mean dissatisfaction score (MDS) was employed as the primary outcome variable. MDS was calculated using TNI values and subsequently categorized into four distinct levels: low, some, moderate, and extreme dissatisfaction. In the analysis of questionnaire survey outcomes, the model was adjusted for the outcome variable "perceived annoyance," which was classified into five categories (not at all, slightly, moderately, very, and extremely annoyed).

In consonance with extant literature and research objectives, 11 predictor variables were identified, encompassing demographic attributes, exposure duration, awareness, and perceived health impacts related to noise pollution. These variables included: gender (male, female), age group (<20, 20–30, 30–40, 40–50, >50), occupational category (driver, shopkeeper, hawker, student, medic, service holder, police, teacher, others, unemployed), level of education (undergraduate, higher secondary, secondary, primary or no education), duration of stay around a traffic intersection (<1, 1–2, 3–4, 5–6, or >6 h), perceived peak noise intensity period (morning, midday, afternoon), perception of increased noise pollution during peak tourist season (yes, no), perceived intensity of noise pollution at traffic intersections (not at all, slightly, moderately, very, and extremely noisy), awareness of health risks associated with excessive noise pollution (yes, no), experienced health consequences due to noise pollution exposure (annoyance, headache, cognitive impairment, hearing difficulty, fatigue, loss of sleep, high blood pressure), and perceived sources of noise pollution at road intersections (vehicle horns, engine sound, crowd of people, miking or speakers, construction works). For the investigation of noise-induced annoyance (MDS), three predictor variables were determined: weekdays (Friday-Thursday), time slots (morning, midday, afternoon), and intersections (BT, HC, KC, LR, BG).

**Fig. 3:**
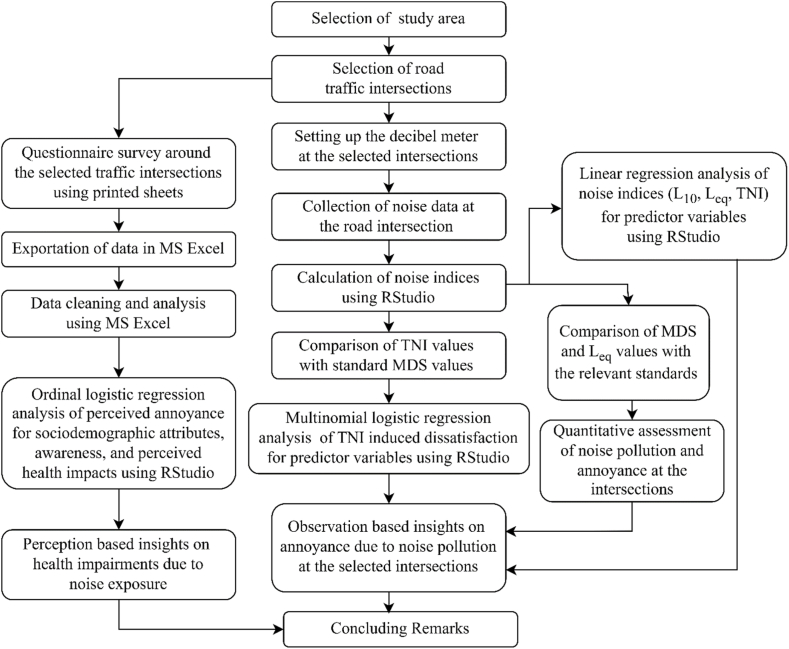
Methodological flow chart of the current study.

An unweighted bivariate analysis was performed to examine the distribution of sociodemographic variables across outcome variables. Associations between perceived annoyance and sociodemographic factors were evaluated using chi-square tests or analysis of variance, contingent upon the variable's measurement scale and category count. Given the four-level categorization of mean dissatisfaction scores, a multinomial logistic regression model was employed to assess associations between TNI-related dissatisfaction and predictor variables. Concurrently, linear regression models were applied to noise indices (L_10_, L_eq_, and TNI). An ordinal logistic regression model for perceived annoyances was adjusted for socio-demographic predictors, perceptions, and health consequences. Uncertainty of estimates was quantified using 95% confidence intervals for regression coefficients and odds ratios, accounting for sampling variability and model errors, but not for measurement errors. All statistical analyses were conducted using SPSS (v.29) and R Language (v.4.2.0), ensuring robust and comprehensive data interpretation. The complete methodological process of the current study is showcased in Fig. 3.

## 3 Results and discussion

Table 1 represents the results of the linear regression analysis model for the key noise indices (L_10_, L_eq_ and TNI) adjusted for three predictors including time slots, weekdays, and road intersections. These statistical analyses complemented the descriptive data, offering a more nuanced understanding of noise pollution patterns in the studied intersections of Cox's Bazar.

**Table 1:** Results of linear regression models for L_10_, L_eq_, and TNI after adjusting for various predictors.

Predictors	Mean (SD)			Regression Coefficients (95% CI)		
	L_10_ (dB)	L_eq_ (dB)	TNI (dB)	L_10_ (R^2^ = 0.470)^a^	L_eq_ (R^2^ = 0.448)^a^	TNI (R^2^ = 0.135)
Time slots						
08 a.m. to 12 p.m.	89.5 (3.3)	86.4 (3.3)	85.2 (8.8)	Reference	Reference	Reference
12 p.m.–04 p.m.	89.5 (2.9)	87.0 (3.2)	87.8 (11.4)	−0.02 (−1.24, 1.2)	0.54 (−0.70, 1.78)	2.63 (−2.4, 7.71)
04 p.m.–08 p.m.	90.6 (3.7)	87.3 (3.4)	88.6 (12.0)	1.15 (−0.07, 2.37)	0.86 (−0.38, 2.11)	3.37 (−1.7, 8.44)
Weekdays						
Friday	89.2 (2.5)	86.8 (2.6)	86.8 (10.8)	Reference	Reference	Reference
Saturday	89.5 (3.1)	86.6 (3.4)	91.0 (11.0)	0.27 (−1.6, 2.13)	−0.20 (−2.10, 1.70)	4.2 (−3.6, 11.95)
Sunday	89.6 (4.0)	86.8 (4.4)	85.2 (11.9)	0.42 (−1.45, 2.28)	0.06 (−1.84, 1.95)	−1.52 (−9.3, 6.24)
Monday	89.2 (3.9)	86.1 (4.0)	83.4 (9.6)	−0.01 (−1.87, 1.85)	−0.65 (−2.54, 1.25)	−3.38 (−11.1, 4.38)
Tuesday	90.5 (3.2)	87.3 (3.1)	87.0 (9.5)	1.33 (−0.54, 3.19)	0.54 (−1.35, 2.44)	0.29 (−7.5, 8.04)
Wednesday	90.7 (3.9)	87.0 (3.2)	90.9 (12.3)	1.46 (−0.40, 3.32)	0.22 (−1.68, 2.12)	4.18 (−3.6, 11.94)
Thursday	90.4 (2.5)	87.7 (2.5)	86.0 (9.3)	1.15 (−0.71, 3.02)	0.96 (−0.93, 2.86)	−0.77 (−8.5, 6.98)
Road Intersections						
Bus Terminal	90.3 (3.1)	87.6 (3.1)	87.1 (13.5)	Reference	Reference	Reference
Kolatoli Circle	91.5 (2.8)	88.6 (2.7)	88.6 (10.9)	1.2 (−0.38, 2.77)^b^	0.97 (−0.63, 2.58)	1.53 (−5, 8.08)
Link Road	92.7 (2.5)	89.5 (2.5)	90.8 (10.5)	2.38 (0.81, 3.96)^b^	1.80 (0.20, 3.41)^b^	3.73 (−2.8, 10.28)
Holiday Circle	87.2 (1.5)	84.1 (2.1)	86.5 (9.7)	−3.06 (−4.63, −1.48)	−3.55 (−5.16, −1.95)^b^	−0.53 (−7.1, 6.02)
Bazar Ghata	87.7 (2.8)	84.8 (2.5)	83.0 (8.0)	−2.62 (−4.19, −1.04)^b^	−2.89 (−4.5, −1.29)^b^	−4.08 (−10.6, 2.47)

a Model Significance at p < 0.05

b Predictor Significance at p < 0.05

Significant variations in noise levels and noise dissatisfaction were revealed across different time slots, days of the week, and road intersections. Consistent higher levels were observed in the afternoon time slot compared to the morning, with a regression coefficient of 1.15 dB (95% CI: 0.07, 2.37) being recorded, which indicates a consistent elevation in peak noise levels during the afternoon hours. Similarly, elevated L_eq_ (β = 0.86, 95% CI: 0.38, 2.11) and TNI (β = 3.37, 95% CI: 1.7, 8.44) values were noted in the afternoon, which suggest a general trend of increased noise pollution as the day progresses, potentially due to accumulating traffic and human activities. In Dehradun city, Uttarakhand, the highest noise pollution levels were recorded in the afternoon during the summer season, attributed to the influx of tourists and increased vehicular traffic [100]. Similar findings have been reported in other major cities of developing countries [101,102].

Regarding weekday variations, highest L_10_ levels were exhibited on the Wednesdays compared to Friday (β = 1.46, 95% CI: 0.40, 3.32), followed by Tuesday (β = 1.33, 95% CI: 0.54, 3.19). This pattern suggests that midweek days may experience more intense noise peaks, possibly due to increased commercial and tourist activities. L_eq_ values showed less pronounced variations across weekdays, with Thursday exhibiting the highest levels compared to Friday (β = 0.96, 95% CI: 0.93, 2.86). TNI values were found to be highest on Saturday (β = 4.2, 95% CI: 3.6, 11.95) and Wednesday (β = 4.18, 95% CI: 3.6, 11.94) compared to Friday. Overall, most weekdays had a higher likelihood of elevated noise indices compared to Fridays, as also noted in previous studies [101,103].

The analysis of road intersections yielded substantial spatial variability, with certain intersections emerging as noise hotspots. Link Road consistently showed higher L_10_, L_eq_, and TNI values compared to the Bus Terminal, with regression coefficients of 2.38 dB (95% CI: 0.81, 3.96), 1.80 dB (95% CI: 0.20, 3.41), and 3.73 dB (95% CI: 2.8, 10.28), respectively. Conversely, Holiday Circle and Bazar Ghata exhibited lower L_10_, L_eq_, and TNI values compared to the Bus Terminal.

**Fig. 4:**
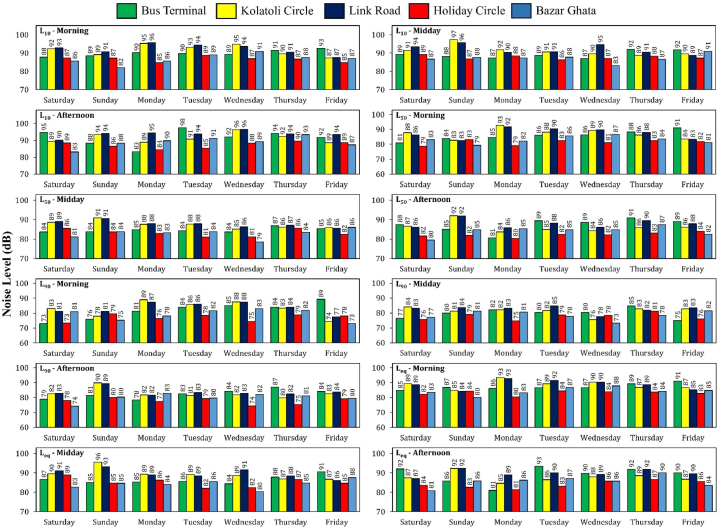
Noise indices by time slot across five intersections over seven weekdays.

The recorded noise levels significantly exceeded permissible limits across all intersections throughout the entire week and all three designated time slots, with consistent patterns of elevated levels particularly during weekends and tourist-heavy periods. The observed noise percentile values from the real-time field data collection are shown in Table S4 and Fig. 4. The Department of Environment (DoE) in Bangladesh established the permissible thresholds for L_eq_ values in commercial and industrial areas at 70 dB and 75 dB, respectively (Table S1). The L_eq_ values exceeded the DOE-mandated threshold by margins ranging from 107% to 127% at the various intersections (Table S2), which suggest a strong correlation between increased human activity and higher noise pollution, highlighting the need for targeted noise management strategies in these high-traffic areas.

Noise levels at the Bus Terminal Intersection exhibited temporal variations throughout the week. Peak values were recorded on Friday mornings (L_10_: 89 dB, L_eq_: 91 dB), potentially due to increased weekend tourist activity. Thursday midday periods demonstrated the most significant acoustic fluctuations (L_10_: 92 dB, L_eq_: 88 dB), while Saturday afternoons showed notably elevated noise levels (L_eq_: 91 dB) compared to other weekdays. At the Link Road intersection, Monday mornings presented the highest noise levels (L_10_: 96 dB, L_eq_: 91 dB). Tuesdays and Sundays exhibited elevated noise indices during daytime and afternoon periods relative to other weekdays, with midday measurements of L_10_: 96 dB and L_eq_: 93 dB, and afternoon levels of L_10_: 94 dB and L_eq_: 92 dB. Kolatoli Circle Intersection displayed noise patterns similar to Link Road Intersection, with peak levels during midday and afternoon periods. Midday measurements reached L_10_: 97 dB and L_eq_: 96 dB, while afternoon levels were L_10_: 96 dB and L_eq_: 92 dB. This pronounced noise pollution on Sunday midday and afternoon may be attributed to concentrated tourist activities. Monday mornings also demonstrated elevated noise indices (L_10_: 95 dB, L_eq_: 93 dB). At Holiday Circle Intersection, Sunday mornings recorded L_10_: 89 dB and L_eq_: 84 dB. Saturday midday periods exhibited L_10_: 89 dB and L_eq_: 89 dB. Friday afternoons showed the highest noise levels (L_10_: 97 dB, L_eq_: 96 dB), potentially influenced by weekend tourist activities. Bazar Ghata Intersection displayed varying noise levels across different time periods. Wednesday mornings showed relatively higher levels (L_10_: 91 dB, L_eq_: 88 dB). Friday daytime hours exhibited L_10_: 91 dB and L_eq_: 88 dB, while Tuesday afternoons recorded the highest levels (L_10_: 93 dB, L_eq_: 90 dB).

Study also revealed concerning noise conditions that have been frequently overlooked, raising significant issues regarding the resulting dissatisfaction. The results from the multinomial regression model for TNI associated dissatisfaction are presented in Table 2. At Link Road, the likelihood of experiencing high and extreme dissatisfaction was elevated compared to the Bus Terminal, with odds ratios of 10.76 (95% CI: 0.77, 151.18) and 6.3 (95% CI: 0.45, 89), respectively. Kolatoli Circle also showed increased odds of high and extreme dissatisfaction (OR = 10.73, 95% CI: 0.77, 150.42 and OR = 3.99, 95% CI: 0.27, 59.7, respectively). This aligns with the higher noise levels observed at Link Road and suggests that Kolatoli Circle, despite not having the highest measured noise levels, can be perceived as particularly annoying. Temporal patterns in dissatisfaction were also observed. The midday time slot was associated with increased odds of moderate (OR = 5.09, 95% CI: 0.83, 31.31) and extreme (OR = 6.28, 95% CI: 0.75, 52.53) dissatisfaction compared to the morning. Interestingly, while the afternoon time slot showed higher noise levels in the L_10_, L_eq_, and TNI analyses, it corresponded to lower odds of high dissatisfaction (OR = 0.67, 95% CI: 0.12, 3.77) but higher odds of extreme dissatisfaction (OR = 4.54, 95% CI: 0.72, 28.56) compared to the morning across the intersections. Overall, midday and afternoon timeslots showed higher likelihood of have higher dissatisfaction compared to morning, which was also evident in other acoustic studies [104,105]. Moreover, Saturday exhibited the highest odds of extreme dissatisfaction (OR = 8.96, 95% CI: 0.49, 164.63) compared to Friday, followed closely by Wednesday (OR = 8.94, 95% CI: 0.48, 165.77). This trend might be related to increased weekend activities or changes in noise tolerance during leisure time.

**Table 2:** Multinomial regression results for mean dissatisfaction scores (MDS) adjusted for predictors.

Predictors	TNI associated dissatisfaction (MDS)			
	Some	Moderate	High	Extreme
Timeslots				
08 a.m. to 12 p.m.	1 (Reference)	1 (Reference)	1 (Reference)	1 (Reference)
12 p.m.–04 p.m.	1 (Reference)	5.09 (0.83, 31.31)	1.64 (0.23, 11.58)	6.28 (0.75, 52.53)
04 p.m.–08 p.m.	1 (Reference)	1.94 (0.42, 9.03)	0.67 (0.12, 3.77)	4.54 (0.72, 28.56)
Weekdays				
Friday	1 (Reference)	1 (Reference)	1 (Reference)	1 (Reference)
Saturday	1 (Reference)	4.19 (0.28, 61.88)	2.08 (0.14, 31.02)	8.96 (0.49, 164.63)
Sunday	1 (Reference)	1.8 (0.22, 14.8)	0.45 (0.05, 4.25)	0.98 (0.07, 13.85)
Monday	1 (Reference)	3.23 (0.34, 30.35)	0.7 (0.06, 7.58)	1.54 (0.1, 24.54)
Tuesday	1 (Reference)	4.87 (0.34, 69.52)	3.81 (0.28, 52.04)	1.58 (0.05, 46.72)
Wednesday	1 (Reference)	6.03 (0.42, 85.98)	0.96 (0.05, 17.25)	8.94 (0.48, 165.77)
Thursday	1 (Reference)	8.76 (0.63, 122.04)	0.02 (0, 0.03)	6.91 (0.36, 133.38)
Intersections				
Bus Terminal	1 (Reference)	1 (Reference)	1 (Reference)	1 (Reference)
Kolatoli Circle	1 (Reference)	3.6 (0.31, 41.41)	10.73 (0.77, 150.42)	3.99 (0.27, 59.7)
Link Road	1 (Reference)	2.81 (0.24, 33.26)	10.76 (0.77, 151.18)	6.3 (0.45, 89)
Holiday Circle	1 (Reference)	1.05 (0.16, 6.71)	2.37 (0.28, 20.27)	1.67 (0.2, 14.08)
Bazar Ghata	1 (Reference)	1.8 (0.3, 10.96)	1.35 (0.14, 12.94)	0.66 (0.06, 7.19)

**Fig. 5:**
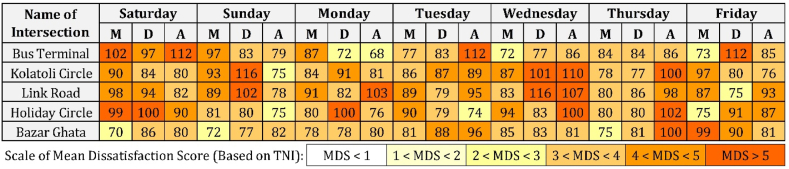
TNI-associated MDS by timeslots across five intersections over seven weekdays.

Fig. 5 presents TNI and the corresponding MDS values for all five intersections across three daily time slots for each day of the week, totaling 105 observations. At the Bus Terminal intersection, extreme dissatisfaction was prevalent throughout Saturdays, with noise levels ranging from 77 dB to 112 dB. Fridays exhibited high dissatisfaction during middays and afternoons (85–112 dB), while Monday daytime and afternoon, and Wednesday and Friday mornings showed low dissatisfaction. Kolatoli Circle consistently demonstrated dissatisfaction levels above the low threshold. TNI values ranged from a low of 77 dB on Thursday midday to a peak of 116 dB on Sunday midday. Link Road recorded its lowest TNI (78 dB) on Sunday afternoons and highest (116 dB) on Wednesday middays. Most time slots (9 out of 21) at Link Road exhibited moderate to high dissatisfaction, with only Friday middays falling within the low dissatisfaction range. Holiday Circle predominantly experienced moderate to high dissatisfaction, with TNIs varying from 74 dB to 102 dB. Extreme dissatisfaction was observed during Saturday mornings (99 dB) and middays (100 dB), Thursday afternoons (102 dB), Wednesday afternoons (100 dB), and Monday middays (100 dB). Bazar Ghata Intersection demonstrated the most consistent moderate dissatisfaction levels, with 13 out of 21 time slots falling in this range. Maximum TNIs were recorded on Thursday afternoons (100 dB) and Friday mornings (99 dB). Low dissatisfaction was noted during Saturday (70 dB), Sunday (72 dB), and Thursday (75 dB) morning slots. Analysis reveals that TNIs frequently exceeded standard dissatisfaction regulations (Table S2) across all intersections and time slots, ranging from 68 to 112 dB. No intersection recorded observations below the low dissatisfaction threshold, despite annoyance standards mandating TNIs below 76 dB. Several intersections consistently exhibited TNIs surpassing the high dissatisfaction level, indicating potential extreme noise dissatisfaction throughout all locations and time periods.

Certain intersections demonstrated consistently higher TNI values, suggesting greater susceptibility to noise issues. Extreme dissatisfaction was regularly observed at the Bus Terminal on Saturdays, Kolatoli Circle on Wednesdays, Link Road on Saturdays and Sundays, Holiday Circle on Saturdays, and Bazar Ghata on Fridays. Multiple time slots across all intersections exceeded the extreme dissatisfaction threshold. Notably, 48 out of 105 time slots exhibited moderate dissatisfaction, distributed as follows: Bazar Ghata (13), Kolatoli Circle (11), Bus Terminal (10), and both Link Road and Holiday Circle (7 each).

Previous studies have documented significant increases in noise pollution and resulting dissatisfaction due to mass tourism in popular destinations [37,[106], [107], [108], [109], [110]]. Research in Madhya, India, indicated noise levels exceeding 80 dB in nearby villages during peak tourist seasons [111]. A study in Kathmandu revealed maximum and average L_eq_ levels of 104 dB and 76 dB, respectively, in high-traffic density areas [112]. Another study in Puri, India, identified elevated noise levels (L_max_ = 98.3 dB, L_eq_ = 80.7 dB) during afternoon hours [113].

The Bus Terminal and Link Road intersections function as essential transportation centers, facilitating both inter-city and local bus services that connect Cox's Bazar City with other districts in Bangladesh and significant tourist spots such as Saint-Martin and Teknaf. The steady stream of tourists, coupled with activities like luggage handling, vehicle operations, and the presence of local CNGs, taxis, and rickshaws, along with the disorganized placement of shops and markets, greatly contributes to increased noise levels in these areas. This issue is particularly noticeable on Saturdays, when tourist traffic peaks, causing a surge in noise annoyance. The continuous noise at these terminals can pose a consistent disturbance to passengers, staff, and nearby residents [35]. Additionally, it has been reported that noise pollution in transportation infrastructure leads to various adverse health effects, such as irritation, reduced work capacity, poor health, and diminished quality of life post-work, though these impacts are still not well-explored in Bangladesh and several other neighboring countries [35,114].

The Kolatoli Circle and Holiday Circle intersections serve as vital junctions connecting various tourist spots in Cox's Bazar City. Holiday Circle, situated en route to the Cox's Bazar airport and the picturesque island of Maheshkhali, faced heightened noise pollution. Its proximity to the sea fish ghat further exacerbated the situation as activities related to the fishing industry generated substantial noise, creating an incessant soundscape of engines and machinery. Kolatoli Circle marks the starting point of the renowned Marine Drive Road, a popular tourist route [115]. Additionally, Kolatoli Circle, adjacent to several large hotels where tourists often stay, could experience a substantial rush of visitors, further intensifying noise levels [116]. The choice of Wednesdays and Saturdays for recording higher TNI values might be due to these days aligning with peak tourist arrivals and activities.

Bazar Ghata, another intersection with consistently high TNI values, serves as a major industrial center flanked by commercial districts and local marketplaces, including the region's largest Burmese markets. The area's vibrant economic activity, particularly on Fridays when both residents and tourists flock to shop, exacerbates traffic congestion on surrounding roads, likely contributing to elevated noise levels. The concentration of street vendors, shopkeepers, and customers in close proximity likely intensifies noise pollution at this intersection, a phenomenon observed in other urban centers [[117], [118], [119]]. Furthermore, the presence of factories, significant hospitals, and clinics could contribute to the persistent noise pollution in this part.

The administered questionnaire survey unveiled some key insights about local residents' perceptions of noise pollution at the urban intersections, its potential contributing factors, and associated health impacts. The general demographic profile of the survey respondents and the summary of the survey adjusted ordinal logistic regression model are presented in Table 3. The survey revealed diverse demographic characteristics among the respondents with the majority respondents (82.70%) being male, and females constituting 17.30% of the sample. A slightly higher likelihood of increased annoyance was observed among females (aOR = 1.19, 95% CI: 0.67, 2.14) compared to males, although this difference was not statistically significant. Age distribution was varied, with the largest group being 31–40 years (29.33%), followed by 21–30 years (23.81%). The mean age of the respondents was 33.37 years. In terms of annoyance levels, a significantly lower odds of high annoyance were demonstrated by the 41–50 age group (aOR = 0.48, 95% CI: 0.23, 0.99) compared to those over 50 years old. This finding suggests potential age-related differences in noise tolerance or perception.

**Table 3:** Respondents' perceived levels of annoyance by socio-demographic attributes, exposure duration, awareness, and perceived health impacts related to noise pollution.

Demographic Characteristics	Level of Annoyance					Total (N)	aOR (95% CI)
	Extremely Annoyed	Very Annoyed	Moderately Annoyed	Slightly Annoyed	Not At All Annoyed		
Sex Comparison (χ^2^ Test Significance = 0.258)							
Female	32 (4.74)	36 (5.33)	26 (3.85)	18 (2.67)	05 (0.74)	117 (17.33)	1.19 (0.67, 2.14)
Male	145 (21.48)	187 (27.7)	121 (17.93)	81 (12)	24 (3.56)	558 (82.67)	1 (Reference)
Age Comparison (χ^2^ Test Significance = 0.988)							
Less than 20	25 (3.7)	35 (5.19)	22 (3.26)	20 (2.96)	01 (0.15)	103 (15.26)	1.32 (0.33, 5.33)
21–30	54 (8)	60 (8.89)	44 (6.52)	20 (2.96)	06 (0.89)	184 (27.26)	0.94 (0.34, 2.57)
31–40	51 (7.56)	61 (9.04)	45 (6.67)	33 (4.89)	08 (1.19)	198 (29.33)	1.02 (0.46, 2.26)
41–50	28 (4.15)	44 (6.52)	25 (3.7)	18 (2.67)	12 (1.78)	127 (18.81)	0.48 (0.23, 0.99)
More than 50	19 (2.81)	23 (3.41)	11 (1.63)	08 (1.19)	02 (0.3)	63 (9.33)	1 (Reference)
Occupational Comparison (χ^2^ Test Significance = 0.264)							
Driver	45 (6.67)	55 (8.15)	39 (5.78)	27 (4.00)	08 (1.19)	174 (25.78)	1.20 (0.26, 5.63)
Shopkeeper	40 (5.93)	33 (4.89)	20 (2.96)	11 (1.63)	03 (0.44)	107 (15.85)	2.47 (0.70, 8.75)
Hawker	18 (2.67)	38 (5.63)	18 (2.67)	09 (1.33)	05 (0.74)	88 (13.04)	1.36 (0.22, 8.62)
Student	20 (2.96)	20 (2.96)	21 (3.11)	13 (1.93)	03 (0.44)	77 (11.41)	0.70 (0.24, 2.04)
Medic	22 (3.26)	17 (2.52)	12 (1.78)	11 (1.63)	02 (0.30)	64 (9.48)	0.98 (0.26, 3.63)
Service Holder	11 (1.63)	14 (2.07)	18 (2.67)	10 (1.48)	04 (0.59)	57 (8.44)	0.85 (0.22, 3.33)
Police	08 (1.19)	19 (2.81)	09 (1.33)	09 (1.33)	04 (0.59)	49 (7.26)	0.88 (0.23, 3.42)
Teacher	08 (1.19)	13 (1.93)	04 (0.59)	04 (0.59)	00 (0.00)	29 (4.30)	1.14 (0.25, 5.25)
Others	01 (0.15)	03 (0.44)	02 (0.30)	03 (0.44)	00 (0.00)	09 (1.33)	1.79 (0.23, 13.7)
Unemployed	04 (0.59)	11 (1.63)	04 (0.59)	02 (0.30)	00 (0.00)	21 (3.11)	1 (Reference)
Educational Comparison (χ^2^ Test Significance = 0.614)							
No education	06 (0.89)	10 (1.48)	09 (1.33)	06 (0.89)	00 (0.00)	31 (4.59)	0.51 (0.08, 3.30)
Primary	37 (5.48)	56 (8.30)	27 (4.00)	16 (2.37)	05 (0.74)	141 (20.89)	0.38 (0.09, 1.62)
Secondary	45 (6.67)	46 (6.81)	38 (5.63)	25 (3.7)	09 (1.33)	163 (24.15)	0.54 (0.19, 1.50)
Higher Secondary	33 (4.89)	51 (7.56)	40 (5.93)	24 (3.56)	07 (1.04)	155 (22.96)	0.48 (0.25, 0.92)
Undergraduate	56 (8.30)	60 (8.89)	33 (4.89)	28 (4.15)	08 (1.19)	185 (27.41)	1 (Reference)
Hours of stay in or around a traffic intersection (χ^2^ Test Significance = 0.000)							
Less than one	04 (0.59)	25 (3.7)	46 (6.81)	67 (9.93)	19 (2.81)	161 (23.85)	0.14 (0.07, 0.26)^a^
One to two	06 (0.89)	33 (4.89)	61 (9.04)	20 (2.96)	09 (1.33)	129 (19.11)	0.10 (0.05, 0.18)^a^
Three to four	33 (4.89)	47 (6.96)	13 (1.93)	07 (1.04)	01 (0.15)	101 (14.96)	1 (Reference)
Five to six	47 (6.96)	37 (5.48)	12 (1.78)	00 (0.00)	00 (0.00)	96 (14.22)	2.52 (1.33, 4.79)^a^
More than six	87 (12.89)	81 (12)	15 (2.22)	05 (0.74)	00 (0.00)	188 (27.85)	2.48 (1.41, 4.36)^a^
Do you think noise pollution increases during the peak tourist season? (χ^2^ Test Significance = 0.483)							
Yes	170 (25.19)	212 (31.41)	142 (21.04)	91 (13.48)	27 (4.00)	642 (95.11)	0.70 (0.32, 1.52)
No	07 (1.04)	11 (1.63)	05 (0.74)	08 (1.19)	02 (0.3)	33 (4.89)	1 (Reference)
What is the intensity of noise pollution in these traffic intersections? (χ^2^ Test Significance = 0.941)							
Extremely noisy	69 (10.22)	95 (14.07)	64 (9.48)	37 (5.48)	14 (2.07)	279 (41.33)	1.04 (0.53, 2.06)
Very noisy	85 (12.59)	96 (14.22)	68 (10.07)	47 (6.96)	13 (1.93)	309 (45.78)	1.12 (0.57, 2.19)
Moderately noisy	14 (2.07)	16 (2.37)	11 (1.63)	09 (1.33)	02 (0.30)	52 (7.70)	1 (Reference)
Slightly noisy	03 (0.44)	04 (0.59)	02 (0.30)	01 (0.15)	00 (0.00)	10 (1.48)	1.12 (0.24, 5.18)
Not at all noisy	06 (0.89)	12 (1.78)	02 (0.30)	05 (0.74)	00 (0.00)	25 (3.70)	1.99 (0.67, 5.88)
At which time intensity of noise pollution is the most at these road intersections? (χ^2^ Test Significance = 0.385)							
04 p.m.–08 p.m.	106 (15.70)	112 (16.59)	85 (12.59)	51 (7.56)	19 (2.81)	373 (55.26)	0.66 (0.44, 0.99)
12 p.m.–04 p.m.	29 (4.30)	52 (7.70)	29 (4.30)	17 (2.52)	05 (0.74)	132 (19.56)	0.63 (0.38, 1.03)
08 a.m.–12 p.m.	42 (6.22)	59 (8.74)	33 (4.89)	31 (4.59)	05 (0.74)	170 (25.19)	1 (Reference)
Do you know excessive noise pollution can threaten your physical or mental well-being? (χ^2^ Test Significance = 0.000)							
Yes	139 (20.59)	206 (30.52)	147 (21.78)	99 (14.67)	29 (4.3)	620 (91.85)	0.02 (0.01, 0.05)^a^
No	38 (5.63)	17 (2.52)	00 (0.00)	00 (0.00)	00 (0.00)	55 (8.15)	1 (Reference)
What health consequences do you face while being exposed noise pollution at these intersections? (χ^2^ Test Significance = 0.000)							
Annoyance	177 (26.22)	223 (33.04)	147 (21.78)	23 (3.41)	00 (0.00)	570 (84.44)	3E9 (3E9, E39)
Headache	105 (15.56)	143 (21.19)	91 (13.48)	62 (9.19)	20 (2.96)	421 (62.37)	0.86 (0.61, 1.22)
Cognitive impairment	80 (11.85)	97 (14.37)	68 (10.07)	42 (6.22)	13 (1.93)	300 (44.44)	0.78 (0.45, 1.33)
Hearing difficulty	78 (11.56)	96 (14.22)	63 (9.33)	40 (5.93)	11 (1.63)	288 (42.67)	1.42 (0.79, 2.55)
Fatigue	56 (8.30)	72 (10.67)	49 (7.26)	31 (4.59)	12 (1.78)	220 (32.59)	0.92 (0.64, 1.32)
Loss of sleep	22 (3.26)	40 (5.93)	19 (2.81)	14 (2.07)	04 (0.59)	99 (14.67)	1.15 (0.71, 1.87)
High blood pressure	65 (9.63)	00 (0.00)	00 (0.00)	00 (0.00)	00 (0.00)	65 (9.63)	1E9 (3E7, 3E10)
What are the possible sources of noise pollution at these road intersections? (χ^2^ Test Significance = 0.000)							
Vehicle horns	177 (26.22)	198 (29.33)	147 (21.78)	99 (14.67)	29 (4.3)	650 (96.30)	0.68 (0.30, 1.56)
Engine sound	100 (14.81)	125 (18.52)	75 (11.11)	60 (8.89)	15 (2.22)	375 (55.56)	1.12 (0.80, 1.58)
Crowd of people	92 (13.63)	111 (16.44)	77 (11.41)	53 (7.85)	12 (1.78)	345 (51.11)	1.08 (0.77, 1.52)
Miking or speakers	85 (12.59)	112 (16.59)	76 (11.26)	52 (7.70)	19 (2.81)	344 (50.96)	0.99 (0.70, 1.39)
Construction works	34 (5.04)	57 (8.44)	41 (6.07)	28 (4.15)	02 (0.30)	162 (24.00)	0.98 (0.55, 1.75)

a Predictor significance at p < 0.05.

Occupationally, the sample included a significant proportion of drivers group consisting of rickshaw-pullers, bus drivers, taxi drivers (25.78%), shop owners and vendors (15.85%), and laborers (13.04%), among others. The highest likelihood of increased annoyance was reported by shopkeepers (aOR = 2.47, 95% CI: 0.70, 8.75), followed by hawkers (aOR = 1.36, 95% CI: 0.22, 8.62) and drivers (aOR = 1.20, 95% CI: 0.26, 5.63), compared to the unemployed reference group. Educational background varied among respondents, with 27.41% holding at least an undergraduate degree. Interestingly, individuals with higher secondary education reported significantly lower odds of high annoyance (aOR = 0.48, 95% CI: 0.25, 0.92) compared to those with undergraduate degrees, warranting further investigation into the role of education in noise perception and tolerance.

The duration of exposure to traffic intersection noise emerged as a highly significant predictor of annoyance levels (p < 0.05). A notable proportion of respondents (27.85%) reported staying at these intersections for more than 6 h daily. These individuals showed significantly higher odds of experiencing extreme annoyance (aOR = 2.48, 95% CI: 1.41, 4.36) compared to those with three to 4 h of exposure. Similarly, those exposed for five to 6 h also reported significantly higher annoyance levels (aOR = 2.52, 95% CI: 1.33, 4.79). Conversely, shorter exposure durations of less than 1 h (aOR = 0.14, 95% CI: 0.07, 0.26) and one to 2 h (aOR = 0.10, 95% CI: 0.05, 0.18) were associated with significantly lower annoyance levels. These findings strongly suggest a dose-response relationship between noise exposure duration and perceived annoyance in the study region.

The survey also sought to determine the time periods when noise pollution was most pronounced. The majority of respondents (55.26%) identified the evening time slot (04:00 p.m. to 08:00 p.m.) as the period with the highest noise pollution intensity, followed by the morning period from 08:00 a.m. to 12:00 p.m. (25.19%). The midday period (12:00 p.m. to 04:00 p.m.) was reported to have the lowest noise intensity (19.56%).

The vast majority of respondents (95.11%) believed that noise pollution increases during peak tourist seasons. This observation underscored the perceived impact of tourism on noise levels at road intersections. It is necessary to conduct additional research and data analysis to confirm and fully understand this prevalent opinion. Regarding the intensity of noise pollution, 41.48% of respondents perceived the noise as "extremely noisy," while 45.78% considered it "very noisy" during the tourist season. Only a small percentage found the noise to be "moderately noisy" (7.70%), "slightly noisy" (1.48%), or "not at all" (3.56%). A significant association was found between awareness of potential health threats posed by excessive noise pollution and annoyance levels (p < 0.05). Notably, 92.00% of respondents acknowledged the adverse impact of noise pollution on physical or mental well-being. Respondents who were aware of these health risks were significantly less likely to report high annoyance levels (aOR = 0.02, 95% CI: 0.01, 0.05), suggesting that knowledge about noise pollution's impacts might influence individual tolerance or coping mechanisms.

The survey highlighted various health consequences experienced by the respondents. "Annoyance" was reported by a significant majority (84.44%), followed by "headache" (62.37%), "cognitive impairment" (44.44%), "hearing difficulty" (42.67%), "fatigue" (32.59%), "loss of sleep" (14.67%), and "high blood pressure" (9.63%). The ordinal logistic regression analysis revealed significant associations between certain health consequences and reported annoyance levels. Notably, respondents who reported experiencing annoyance as a health consequence had extremely high odds of reporting higher levels of overall annoyance (aOR = 3x10^9^, 95% CI: 3x10^9^, 10^39^). This finding suggests that the experience of annoyance itself is a strong predictor of overall noise-related disturbance. Similarly, those reporting high blood pressure as a consequence also showed significantly higher odds of increased annoyance levels (aOR = 1x10^9^, 95% CI: 3x10^7^, 3x10^10^). These results highlight the potentially severe impact of noise pollution on both psychological and physiological well-being. Other reported health consequences, including headache (aOR = 0.86, 95% CI: 0.61, 1.22), cognitive impairment (aOR = 0.78, 95% CI: 0.45, 1.33), hearing difficulty (aOR = 1.42, 95% CI: 0.79, 2.55), fatigue (aOR = 0.92, 95% CI: 0.64, 1.32), and loss of sleep (aOR = 1.15, 95% CI: 0.71, 1.87), did not show statistically significant associations with overall annoyance levels. However, their prevalence among respondents underscored the wide-ranging health impacts of noise pollution. These findings collectively underscored the multifaceted impact of noise pollution on the well-being of individuals in proximity to these areas. These results highlighted the widespread perception of noise pollution as a considerable source of irritation for individuals residing or working near these intersections. The fact of a substantial portion of the population in this area being affected by these health issues emphasized the urgency of implementing noise reduction measures at these busy traffic intersections. Previous studies have already suggested that noise pollution control can significantly improve overall public health by mitigating widespread annoyance and adverse health effects [33,120,121].

Regarding the sources of noise pollution, respondents predominantly identified "vehicle horns" (96.30%) as the primary source, followed by "engine sound" (55.56%), "crowd of people" (51.26%), "mikes or loudspeakers" (50.96%), and "construction works" (24.00%). The regression analysis for these sources revealed interesting patterns, although not all were statistically significant. Vehicle horns, despite being the most commonly reported source, showed a negative association with annoyance levels (aOR = 0.68, 95% CI: 0.30, 1.56), though this was not statistically significant. This unexpected result might suggest a level of habituation to this ubiquitous noise source. Engine sounds (aOR = 1.12, 95% CI: 0.80, 1.58) and crowds of people (aOR = 1.08, 95% CI: 0.77, 1.52) showed slight positive associations with annoyance levels, while miking or speakers (aOR = 0.99, 95% CI: 0.70, 1.39) and construction works (aOR = 0.98, 95% CI: 0.55, 1.75) had negligible associations. These findings highlight the complex nature of noise perception and annoyance, where the most prevalent sources may not necessarily be the most annoying.

The findings provided a nuanced understanding of the noise pollution patterns observed in the study. They revealed not only the quantitative aspects of noise levels but also offer insights into the subjective experience of noise annoyance. The field study found that afternoon time slots generally had higher noise levels across L_10_, L_eq_, and TNI measurements, also substantiated by the survey results showing that the afternoon time slots had higher odds of extreme dissatisfaction. However, this relationship is not straightforward, as survey respondents also reported significant annoyance during morning hours, despite lower measured noise levels. This discrepancy highlighted the multifaceted nature of noise pollution impacts, suggesting that factors beyond mere decibel levels significantly influence perceived annoyance. These findings aligned with previous research that emphasized the complexity of noise perception [122,123]. Various factors may contribute to this phenomenon, including the specific characteristics of noise sources, individual sensitivity thresholds, and temporal expectations of tranquility [[124], [125], [126]]. For instance, even moderate noise levels during typically quiet morning hours might elicit stronger negative reactions than louder sounds at midday when ambient noise is expected to be higher. This response likely stems from the discrepancy between anticipated and actual sound levels. During quieter periods, even moderate disturbances can be perceived as particularly intrusive and disruptive. In contrast, louder noises occurring at midday often merge with the expected environmental soundscape, thereby diminishing their negative impact on individuals [[127], [128], [129]].

However, while offering valuable insights into noise pollution patterns and public perceptions at five key road intersections in Cox's Bazar, Bangladesh, this study was subject to several limitations that warrant consideration when interpreting the results. Firstly, the reliance on self-reported health assessments might have introduced potential bias, as these subjective evaluations might not have aligned with clinical diagnoses. This discrepancy could have led to over- or underestimation of health impacts associated with noise exposure [130,131]. Secondly, the employment of non-probability sampling techniques, specifically convenience sampling, might have introduced selection bias. Despite the anonymous nature of the survey, respondents might have been inclined to provide socially desirable responses, potentially skewing the data [132,133]. Thirdly, the lack of simultaneous collection of noise indices and respondent questionnaire data presented a challenge in establishing direct associations between objective noise levels and public dissatisfaction. This kind of separation limit the ability to draw direct correlations between measured noise levels and reported discomfort [134,135]. Future research would benefit from concurrent data collection to address this limitation. Lastly, the noise data collection period was confined to a 12-h window (8 a.m.–8 p.m.), potentially overlooking important noise patterns during nighttime hours. A more comprehensive 24-h data collection approach in future studies would provide a more holistic understanding of the noise pollution landscape.

Again, in the regression models, several variables that did not reach statistical significance were retained in the final analyses due to their strong theoretical foundations in noise pollution research and to maintain consistency with previous studies [[136], [137], [138]]. The lack of significance in this specific context does not negate their potential importance in other settings or with larger sample sizes [139]. These non-significant results may be attributed to the unique characteristics of Cox's Bazar as a tourist destination, sample size limitations, or other unaccounted factors specific to the study area. Nonetheless, the models yielded valuable insights into noise pollution patterns and annoyance levels, particularly through significant relationships identified for road intersections and certain demographic factors [86,140].

Despite the limitations, this research provided significant insights into noise pollution perceptions in Cox's Bazar. It highlighted the complex interplay between exposure duration, awareness, and health impacts, emphasizing the need for targeted interventions, especially during peak tourist periods and in areas with prolonged exposure. The identification of specific locations, times, and days with elevated noise levels and public dissatisfaction offers crucial information for developing focused noise reduction strategies, with Link Road and Kolatoli Circle suggested as priority areas for remediation. The study also underscored the importance of integrating both objective measurements and subjective experiences to inform effective mitigation strategies. This comprehensive approach could lead to more effective noise reduction efforts that address both physical and psychological aspects of noise. Further research could explore these relationships more deeply, uncovering innovative insights to improve urban planning and noise management policies.

Overall, the noise policy in Bangladesh, primarily governed by the Noise Pollution (Control) Rules of 2006 [85], established specific decibel limits for various urban zones. However, its implementation has been largely ineffective, with urban areas consistently experiencing noise levels far exceeding legal limits [141]. This lack of efficiency is particularly evident in tourist cities and around intersections, where the policy fails to address modern urban challenges adequately [69]. To mitigate these issues, a multifaceted approach is necessary, encompassing the development of specific regulations for tourist areas, implementation of "quiet zones" around major intersections, installation of noise barriers, studying road network structure, and introduction of smart traffic management systems [14,142,143]. Promoting low-noise vehicles, conducting regular noise mapping, enhancing public education efforts, and establishing dedicated noise complaint systems could significantly improve noise management [144,145]. Additionally, public awareness campaigns and community engagement could play a vital role in fostering responsible behavior among tourists and residents. These targeted solutions, combined with overall policy enhancements and stricter enforcement mechanisms, have the potential to substantially reduce noise pollution in these intersections, particularly during the peak tourist seasons.

## 4 Conclusions

Noise pollution at key traffic intersections in Cox's Bazar has been identified as a significant environmental and public health concern. This study provided a comprehensive analysis of noise levels and the associated annoyance experienced during the peak tourist season, offering critical insights into the scope and impact of traffic noise in the city. The results revealed that noise levels consistently exceeded the national guideline of L_eq_ ≤ 75 dB during morning, midday, and afternoon periods, across both weekdays and weekends. The highest levels of noise were observed on Thursdays through Sundays, with notable increases in annoyance reported particularly during the morning and afternoon hours. Only 12 of the time slots assessed showed noise levels below the threshold, while 19 time slots recorded TNI scores surpassing extreme dissatisfaction, indicating a widespread issue with traffic noise. Survey data demonstrated more than 95% of respondents perceiving a rise in noise pollution during the peak tourist season. Additionally, the survey highlighted several adverse health effects related to noise exposure, including headaches, cognitive decline, auditory difficulties, fatigue, sleep disturbances, and elevated blood pressure. These health impacts were strongly linked to increased levels of annoyance, underscoring the profound effect of traffic noise on residents' well-being.

This investigation underscored the importance of studying road traffic noise, particularly in urban tourist areas where vehicle traffic surged during peak periods. Despite awareness among the local community regarding rising noise levels, the extent of its impact is often underestimated. The direct health consequences associated with traffic noise, as identified through the survey, reflected serious implications for residents' quality of life. The diverse range of respondents and their varying exposure durations illuminated both the prevalence and severity of noise pollution, highlighting the community's awareness of its adverse effects and the urgent need for effective noise mitigation strategies. The generalizability of this study results may be limited to similar urban intersections with comparable noise exposure and demographic profiles. While the findings offered valuable insights into noise pollution impacts within the study area, caution should be taken when applying these results to different settings or populations with varying characteristics.

## Funding Statement

This research work was supported by the Deanship of Scientific Research at King Khalid University under grant number RGP.2/279/45.

## CRediT authorship contribution statement

**Hrithik Nath:** Writing – review & editing, Writing – original draft, Visualization, Validation, Supervision, Software, Resources, Project administration, Methodology, Investigation, Formal analysis, Data curation, Conceptualization. **Sajal Kumar Adhikary:** Writing – review & editing, Writing – original draft, Visualization, Validation, Supervision, Methodology, Formal analysis, Data curation. **Saleh Alsulamy:** Writing – review & editing, Supervision, Resources, Project administration, Methodology, Investigation, Funding acquisition, Data curation. **Abdulla Al Kafy:** Writing – review & editing, Writing – original draft, Validation, Supervision, Resources, Methodology, Investigation, Formal analysis. **Zullyadini A. Rahaman:** Writing – review & editing, Validation, Supervision, Project administration, Methodology, Formal analysis. **Srabanti Roy:** Writing – review & editing, Writing – original draft, Visualization, Methodology, Formal analysis. **Mohammad Iqbal Hossain:** Writing – original draft, Project administration, Formal analysis, Data curation. **Abdulla Al Mamun:** Writing – original draft, Project administration, Formal analysis, Data curation.

## Consent to participate

During the data collection, comprehensive information about survey objectives, methodology, and significance was provided to respondents. Explicit informed consent was obtained verbally, assuring confidentiality and privacy protection. Measures were implemented to prevent any disclosure that could identify participants.

## Ethical approval

During noise data collection and field survey, ethical guidelines were prioritized to ensure minimal disruption. Permission from authorities and property owners was obtained before installing noise monitoring equipment. Adherence to ethical principles, including informed consent, data confidentiality, privacy maintenance, and cultural sensitivity, ensured participant welfare. The conscientious authorities of the University of Creative Technology Chattogram (UCTC) reviewed and approved the study design and methods to ensure that they adhered to the ethical standards and guidelines and that the rights and welfare of the participants were protected (Ref: UCTC/Reg/Off-Order-Notice/2022/06).

## Consent to publish

Respondents were assured of the utmost confidentiality in handling personal information, extending to publication. The commitment to protect identities and prevent disclosure was a key element of the consent to publish, highlighting responsible and considerate data handling in dissemination.

## Availability of data and materials

The data that support the findings of this study are available from the corresponding author [Hrithik Nath, hrithiknath.ce@gmail.com] upon reasonable request.

## Declaration of competing interest

The authors declare that they have no known competing financial interests or personal relationships that could have appeared to influence the work reported in this paper.
