# Supplementary material for: Assessment of index-based traffic noise annoyance level at major road intersections in a tourist city: A case study towards environmental sustainability
Source: Heliyon. 2024 Nov 1;10(21):e40005. doi: 10.1016/j.heliyon.2024.e40005 (PMC11570300; doi:10.1016/j.heliyon.2024.e40005)
Supplement: Multimedia component 1 [file mmc1.docx]

**Assessment of Index-Based Traffic Noise Annoyance Level at Major Road Intersections in a Tourist City: A Case Study towards Environmental Sustainability**

Hrithik Nath^1,2^*, Sajal Kumar Adhikary^2^, Saleh Alsulamy^3^, Abdulla Al Kafy^4^, Zullyadini A. Rahaman^5^, Srabanti Roy^6^, Mohammad Iqbal Hossain^1^, Abdulla Al Mamun^1^

^1^Department of Civil Engineering, University of Creative Technology Chittagong (UCTC), Chattogram, 4212, Bangladesh

^2^Department of Civil Engineering, Khulna University of Engineering & Technology, Khulna, 9203, Bangladesh

^3^Department of Architecture, College of Architecture & Planning, King Khalid University, Abha, 61421, Saudi Arabia

^4^Department of Urban & Regional Planning, Rajshahi University of Engineering & Technology (RUET), Rajshahi, 6204, Bangladesh

^5^Department of Geography & Environment, Faculty of Human Sciences, Sultan Idris Education University, Tanjung Malim, 35900, Malaysia

^6^Department of Public Health, University of Creative Technology Chittagong (UCTC), Chattogram, 4212, Bangladesh

*** Corresponding Author:**

Hrithik Nath

Department of Civil Engineering, University of Creative Technology Chittagong (UCTC), Chattogram, 4212, Bangladesh

Email: hrithiknath.ce@gmail.com

OrcId: 0000-0002-8381-715X

**Supplementary Materials**

**Table S1:** Summary of acceptable daytime noise standards (L_eq_) according to different agencies used in recent studies

| **Area Description** | **Acceptable Noise Limits as L_eq_ (dB)** | | |
| --- | --- | --- | --- |
|  | **DOE** | **AASHTO** | **FHA** |
| Sensitive Areas | 45 | 55-60 | 60 |
| Residential Areas | 50 | 77*, 55** | 77*, 55** |
| Mixed Areas | 60 | 70 | 70 |
| Commercial Areas | 70 | 75 | 75 |
| Industrial Areas | 75 | 75 | 75 |
| *Exterior Environment, ** Interior Environment | | | |

**Table S2:** Upper limits of mean dissatisfaction scores (MDS) for traffic noise index (TNI) [1]

| **Noise**  **Indices** | **Mean Dissatisfaction Score in dB (Level of Dissatisfaction)** | | | | |
| --- | --- | --- | --- | --- | --- |
|  | **Desirable** | |  | **Prohibitive** | |
|  | **MDS 2**  **(Some)** | **MDS 3**  **(Moderate)** |  | **MDS 4**  **(High)** | **MDS 5**  **(Extreme)** |
| TNI | 61 | 76 |  | 87 | 98 |

**Table S3:** Summary of noise indices found from the field observation in the current study

| **Noise Indices** | | **Sat** | | | **Sun** | | | **Mon** | | | **Tue** | | | **Wed** | | | **Thu** | | | **Fri** | | |
| --- | --- | --- | --- | --- | --- | --- | --- | --- | --- | --- | --- | --- | --- | --- | --- | --- | --- | --- | --- | --- | --- | --- |
|  |  | **M** | **D** | **A** | **M** | **D** | **A** | **M** | **D** | **A** | **M** | **D** | **A** | **M** | **D** | **A** | **M** | **D** | **A** | **M** | **D** | **A** |
| Bus Terminal | ***L_10_*** | 88 | 89 | 95 | 89 | 88 | 88 | 90 | 87 | 83 | 90 | 89 | 98 | 89 | 87 | 92 | 91 | 92 | 94 | 93 | 92 | 92 |
|  | ***L_50_*** | 81 | 84 | 88 | 84 | 84 | 85 | 85 | 85 | 81 | 86 | 84 | 89 | 86 | 84 | 89 | 88 | 87 | 91 | 91 | 85 | 89 |
|  | ***L_90_*** | 73 | 77 | 79 | 76 | 80 | 81 | 81 | 82 | 78 | 84 | 80 | 83 | 85 | 80 | 84 | 84 | 85 | 87 | 89 | 75 | 84 |
|  | ***L_MAX_*** | 94 | 94 | 99 | 92 | 92 | 95 | 97 | 93 | 88 | 96 | 92 | 101 | 94 | 100 | 95 | 94 | 99 | 95 | 96 | 92 | 95 |
|  | ***Leq*** | 85 | 87 | 92 | 87 | 85 | 86 | 86 | 85 | 81 | 87 | 86 | 93 | 87 | 84 | 90 | 89 | 88 | 92 | 91 | 91 | 90 |
|  | ***NC*** | 15 | 13 | 16 | 13 | 8 | 7 | 9 | 5 | 5 | 6 | 8 | 15 | 4 | 7 | 8 | 7 | 7 | 7 | 3 | 17 | 8 |
|  | ***L_NP_*** | 99 | 99 | 108 | 99 | 93 | 93 | 95 | 90 | 86 | 92 | 94 | 108 | 91 | 91 | 98 | 97 | 95 | 99 | 94 | 107 | 98 |
|  | ***TNI*** | 102 | 97 | 112 | 97 | 83 | 79 | 87 | 72 | 68 | 77 | 83 | 112 | 72 | 77 | 86 | 84 | 84 | 86 | 73 | 112 | 85 |
| Kolatoli Circle | ***L_10_*** | 92 | 91 | 89 | 89 | 97 | 94 | 95 | 92 | 89 | 93 | 91 | 91 | 95 | 90 | 96 | 90 | 89 | 92 | 87 | 90 | 89 |
|  | ***L_50_*** | 88 | 89 | 87 | 83 | 91 | 92 | 93 | 88 | 84 | 88 | 88 | 85 | 89 | 85 | 84 | 86 | 86 | 86 | 84 | 86 | 86 |
|  | ***L_90_*** | 83 | 84 | 82 | 78 | 81 | 90 | 89 | 82 | 82 | 86 | 82 | 81 | 88 | 76 | 82 | 83 | 83 | 80 | 74 | 83 | 83 |
|  | ***L_MAX_*** | 97 | 97 | 98 | 94 | 99 | 96 | 98 | 93 | 93 | 97 | 91 | 99 | 98 | 91 | 100 | 90 | 90 | 95 | 89 | 91 | 95 |
|  | ***Leq*** | 89 | 90 | 87 | 85 | 96 | 92 | 93 | 89 | 85 | 89 | 89 | 86 | 90 | 89 | 88 | 87 | 87 | 89 | 87 | 87 | 87 |
|  | ***NC*** | 9 | 7 | 7 | 11 | 16 | 4 | 6 | 10 | 7 | 8 | 9 | 9 | 7 | 14 | 14 | 6 | 6 | 13 | 13 | 7 | 6 |
|  | ***L_NP_*** | 98 | 97 | 94 | 96 | 111 | 96 | 100 | 99 | 92 | 97 | 98 | 96 | 98 | 102 | 102 | 93 | 93 | 101 | 100 | 93 | 93 |
|  | ***TNI*** | 90 | 84 | 80 | 93 | 116 | 75 | 84 | 91 | 81 | 86 | 87 | 89 | 87 | 101 | 110 | 78 | 77 | 100 | 97 | 80 | 76 |
| Link Road | ***L_10_*** | 93 | 94 | 90 | 91 | 96 | 94 | 96 | 90 | 95 | 94 | 91 | 94 | 94 | 95 | 96 | 91 | 91 | 94 | 87 | 89 | 94 |
|  | ***L_50_*** | 86 | 89 | 86 | 83 | 91 | 92 | 92 | 88 | 86 | 90 | 88 | 88 | 90 | 86 | 86 | 88 | 87 | 90 | 83 | 86 | 88 |
|  | ***L_90_*** | 81 | 83 | 83 | 81 | 84 | 89 | 87 | 83 | 82 | 86 | 85 | 83 | 88 | 78 | 83 | 84 | 82 | 82 | 77 | 83 | 84 |
|  | ***L_MAX_*** | 98 | 96 | 96 | 94 | 99 | 95 | 99 | 91 | 97 | 98 | 94 | 98 | 98 | 99 | 100 | 92 | 91 | 96 | 90 | 91 | 99 |
|  | ***Leq*** | 89 | 91 | 87 | 84 | 93 | 92 | 93 | 89 | 89 | 92 | 89 | 90 | 90 | 91 | 89 | 89 | 88 | 92 | 85 | 86 | 90 |
|  | ***NC*** | 12 | 10 | 7 | 10 | 12 | 5 | 8 | 7 | 13 | 8 | 6 | 10 | 6 | 17 | 13 | 6 | 9 | 11 | 10 | 5 | 10 |
|  | ***L_NP_*** | 100 | 101 | 94 | 94 | 105 | 97 | 101 | 96 | 102 | 100 | 94 | 100 | 97 | 108 | 103 | 95 | 97 | 103 | 95 | 92 | 99 |
|  | ***TNI*** | 98 | 94 | 82 | 89 | 102 | 78 | 91 | 82 | 103 | 89 | 79 | 95 | 83 | 116 | 107 | 80 | 86 | 98 | 87 | 75 | 93 |
| Holiday Circle | ***L_10_*** | 87 | 89 | 89 | 87 | 87 | 86 | 85 | 88 | 84 | 89 | 86 | 85 | 87 | 87 | 88 | 87 | 88 | 90 | 85 | 87 | 89 |
|  | ***L_50_*** | 79 | 86 | 82 | 83 | 84 | 82 | 79 | 83 | 80 | 83 | 81 | 82 | 81 | 81 | 82 | 83 | 86 | 83 | 82 | 82 | 84 |
|  | ***L_90_*** | 73 | 76 | 78 | 79 | 79 | 80 | 76 | 75 | 77 | 78 | 79 | 79 | 75 | 78 | 74 | 79 | 81 | 75 | 78 | 76 | 79 |
|  | ***L_MAX_*** | 88 | 92 | 90 | 90 | 91 | 88 | 87 | 90 | 87 | 90 | 89 | 87 | 89 | 89 | 93 | 88 | 91 | 97 | 86 | 92 | 92 |
|  | ***Leq*** | 82 | 89 | 84 | 84 | 85 | 83 | 80 | 86 | 81 | 84 | 82 | 83 | 84 | 82 | 86 | 84 | 87 | 87 | 83 | 85 | 86 |
|  | ***NC*** | 14 | 14 | 11 | 8 | 8 | 6 | 8 | 14 | 7 | 10 | 8 | 6 | 12 | 9 | 14 | 8 | 7 | 14 | 7 | 11 | 9 |
|  | ***L_NP_*** | 96 | 102 | 94 | 92 | 93 | 89 | 89 | 100 | 88 | 95 | 90 | 89 | 96 | 91 | 99 | 91 | 94 | 101 | 89 | 96 | 95 |
|  | ***TNI*** | 99 | 100 | 90 | 81 | 80 | 75 | 80 | 100 | 76 | 90 | 79 | 74 | 94 | 83 | 100 | 80 | 80 | 102 | 75 | 91 | 87 |
| Bazar Ghata | ***L_10_*** | 86 | 87 | 83 | 82 | 88 | 88 | 86 | 87 | 90 | 89 | 88 | 91 | 91 | 83 | 89 | 88 | 87 | 93 | 87 | 91 | 87 |
|  | ***L_50_*** | 83 | 81 | 80 | 79 | 84 | 85 | 82 | 83 | 85 | 86 | 84 | 85 | 87 | 79 | 85 | 84 | 84 | 87 | 81 | 86 | 82 |
|  | ***L_90_*** | 81 | 77 | 74 | 75 | 81 | 80 | 78 | 81 | 83 | 82 | 78 | 80 | 83 | 73 | 82 | 82 | 78 | 81 | 73 | 82 | 80 |
|  | ***L_MAX_*** | 89 | 90 | 85 | 85 | 90 | 90 | 90 | 89 | 96 | 91 | 93 | 98 | 93 | 88 | 93 | 89 | 90 | 99 | 88 | 94 | 90 |
|  | ***Leq*** | 83 | 83 | 81 | 80 | 85 | 86 | 83 | 84 | 86 | 87 | 86 | 87 | 88 | 80 | 86 | 84 | 85 | 90 | 85 | 88 | 84 |
|  | ***NC*** | 5 | 10 | 9 | 7 | 6 | 8 | 8 | 7 | 7 | 7 | 10 | 12 | 8 | 10 | 7 | 6 | 8 | 12 | 14 | 10 | 8 |
|  | ***L_NP_*** | 88 | 92 | 90 | 87 | 91 | 94 | 91 | 91 | 93 | 94 | 96 | 99 | 96 | 90 | 93 | 90 | 93 | 102 | 99 | 97 | 91 |
|  | ***TNI*** | 70 | 86 | 80 | 72 | 77 | 82 | 78 | 78 | 80 | 81 | 88 | 96 | 85 | 83 | 81 | 75 | 81 | 100 | 99 | 90 | 81 |
| M=Morning, D=Midday, A=Afternoon | | | | | | | | | | | | | | | | | | | | | | |

**Table S4:** L_eq_ values detected at different intersections during different time slots and comparison with the acceptable limit employed by the Department of Environment (DOE), Bangladesh [2]

| **Day** | **Time Slot** |  | **L_eq_ (Deviation in % from relevant DOE Standard, 75 dB)** | | | | | | | | |
| --- | --- | --- | --- | --- | --- | --- | --- | --- | --- | --- | --- |
|  |  |  | **BT** |  | **KC** |  | **LR** |  | **HC** |  | **BG** |
| Sat | Morning |  | 85 (113%) |  | 89 (119%) |  | 89 (118%) |  | 82 (110%) |  | 83 (111%) |
|  | Midday |  | 87 (115%) |  | 90 (119%) |  | 91 (121%) |  | 89 (119%) |  | 83 (110%) |
|  | Afternoon |  | 92 (123%) |  | 87 (117%) |  | 87 (116%) |  | 84 (111%) |  | 81 (108%) |
| Sun | Morning |  | 87 (116%) |  | 85 (113%) |  | 84 (112%) |  | 84 (112%) |  | 80 (107%) |
|  | Midday |  | 85 (113%) |  | 96 (127%) |  | 93 (124%) |  | 85 (113%) |  | 85 (113%) |
|  | Afternoon |  | 86 (114%) |  | 92 (123%) |  | 92 (123%) |  | 83 (110%) |  | 86 (115%) |
| Mon | Morning |  | 86 (115%) |  | 93 (125%) |  | 93 (124%) |  | 80 (107%) |  | 83 (111%) |
|  | Midday |  | 85 (114%) |  | 89 (119%) |  | 89 (119%) |  | 86 (115%) |  | 84 (112%) |
|  | Afternoon |  | 81 (108%) |  | 85 (113%) |  | 89 (119%) |  | 81 (108%) |  | 86 (115%) |
| Tue | Morning |  | 87 (116%) |  | 89 (119%) |  | 92 (122%) |  | 84 (113%) |  | 87 (116%) |
|  | Midday |  | 86 (114%) |  | 89 (119%) |  | 89 (118%) |  | 82 (109%) |  | 86 (114%) |
|  | Afternoon |  | 93 (125%) |  | 86 (115%) |  | 90 (120%) |  | 83 (111%) |  | 87 (116%) |
| Wed | Morning |  | 87 (116%) |  | 90 (120%) |  | 90 (121%) |  | 84 (112%) |  | 88 (117%) |
|  | Midday |  | 84 (113%) |  | 89 (118%) |  | 91 (122%) |  | 82 (110%) |  | 80 (107%) |
|  | Afternoon |  | 90 (120%) |  | 88 (117%) |  | 89 (119%) |  | 86 (114%) |  | 86 (114%) |
| Thu | Morning |  | 89 (119%) |  | 87 (116%) |  | 89 (118%) |  | 84 (112%) |  | 84 (112%) |
|  | Midday |  | 88 (117%) |  | 87 (116%) |  | 88 (118%) |  | 87 (115%) |  | 85 (113%) |
|  | Afternoon |  | 92 (122%) |  | 89 (118%) |  | 92 (122%) |  | 87 (116%) |  | 90 (120%) |
| Fri | Morning |  | 91 (122%) |  | 87 (116%) |  | 85 (113%) |  | 83 (110%) |  | 85 (113%) |
|  | Midday |  | 91 (121%) |  | 87 (116%) |  | 86 (115%) |  | 85 (113%) |  | 88 (117%) |
|  | Afternoon |  | 90 (120%) |  | 87 (116%) |  | 90 (119%) |  | 86 (114%) |  | 84 (111%) |
| *BT: Bus Terminal, KC: Kolatoli Circle, LR: Link Road, HC: Holiday Circle, BG: Bazar Ghata* | | | | | | | | | | | |


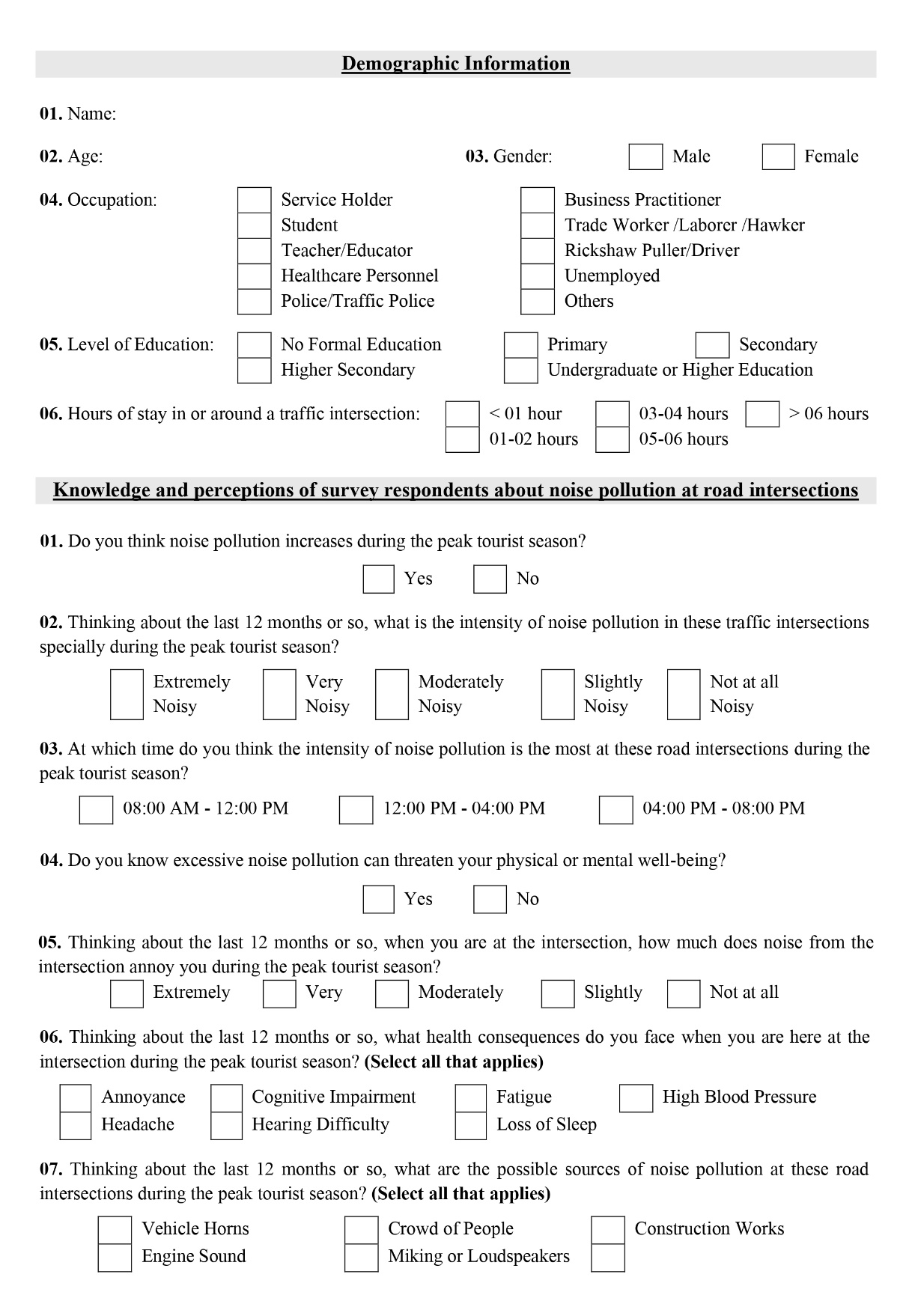


**Fig. S1:** Questionnaire Survey Form

**References**

[1] P.R. Rao, M.G.S. Rao, Community reaction to road traffic noise, Applied Acoustics 37 (1992) 51–64. https://doi.org/10.1016/0003-682X(92)90010-P.

[2] DoE, Department of Environment, (2006). http://www.doe.gov.bd/site/page/77d144abf82a-4fc3-a61c5bd6547ca646/- (accessed August 7, 2024).
